# Systematic review and meta-analysis protocol for efficacy and safety of *Momordica charantia* L. on animal models of type 2 diabetes mellitus

**DOI:** 10.1186/s13643-019-1265-4

**Published:** 2020-01-08

**Authors:** Emanuel L. Peter, Andrew G. Mtewa, Prakash B. Nagendrappa, Anita Kaligirwa, Crispin Duncan Sesaazi

**Affiliations:** 1 Pharm-Biotechnology and Traditional Medicine Center of Excellence (PHARMBIOTRAC), Mbarara University of Science and Technology, P.O. Box 1410, Mbarara, Uganda; 2 Department of Innovation, Technology transfer & Commercialization, National Institute for Medical Research, Dar Es Salaam, Tanzania; 3 Chemistry Section, Department of Applied Sciences, Malawi Institute of Technology, Malawi University of Science & Technology, Limbe, Malawi; 4 Centre for Local Health Traditions & Policy, Trans-disciplinary University (TDU), Bengaluru, India; 5 Department of Pharmacology, Faculty of Medicine, Mbarara University of Science and Technology, Mbarara, Uganda

**Keywords:** Diabetes mellitus type 2, *Momordica charantia*, Animals, Efficacy, Safety, Protocol, Systematic review

## Abstract

**Background:**

Studies on several preclinical models of type 2 diabetes mellitus have been conducted to establish the hypoglycemic activity of *Momordica charantia* L. Concerned with appropriateness of these models, we designed a systematic review to establish the efficacy and safety of *M. charantia* L. in preclinical models of type 2 diabetes mellitus.

**Methods:**

Review authors will search without language restriction in MEDLINE/PubMed, Web of Science, Embase, Scopus, and CINAHL databases through April 2019. Search filters will be applied to enhance search efficiency. The authors will search for gray literature in Google and Google Scholar, OpenGrey, and ProQuest Dissertations & Theses. Two authors will evaluate full texts, extract data, and asses risk of bias independently. The review will include randomized or non-randomized studies that assessed the efficacy or safety of *M. charantia* L. with vehicle control group. The primary endpoint will be fasting blood glucose level. We will use Egger’s test to assess publication biases. Chi-square test and *I*^2^ will be used to assess heterogeneity in effect size of the primary outcome. Using RevMan software version 5.3, the authors will perform a meta-analysis of quantitative data.

**Discussion:**

The strength of evidence will be rated as high, moderate, low, or very low using GRADE framework for animal studies. This systematic review will potentially improve research practice by identifying risks of bias and design features that compromise translatability and contribute to evidence-based clinical trial design.

**Systematic review registration:**

PROSPERO CRD42019119181

## Background

Type 2 diabetes mellitus (T2DM) is a metabolic disorder of multiple physiological abnormalities characterized by chronic hyperglycemia. Studies in pathophysiology and underlying mechanism of diabetes established that the disease process is heterogeneous and at least five pathophysiological abnormalities are involved. Scientists have recognized that insulin resistance mainly in the liver and muscles precedes beta-cell dysfunction [1], progressive defect of beta-cell function and mass, mark manifestation of hyperglycemia and its severity [2]. Other abnormalities include increased lipolysis, and hypothalamic insulin resistance which also impairs the ability of insulin to suppress glucose production, and renal tubular glucose reabsorption capacity [3]. The chronicity of hyperglycemia causes microvascular complications in the retina, renal glomerulus, and peripheral nerves [4] and increases the risk of accelerated atherosclerosis and premature death [5].

In realization of the multiple physiological abnormalities, goals of treatment for type 2 diabetes mellitus are now to prevent or delay complications and maintain quality of life through sustained glycemic control [6]. The American Diabetes Association (ADA) and European Association for the study of diabetes recommend oral hypoglycemic agents (OHAs); biguanides (metformin) as first-line treatment while sulphonylureas, meglitinides (glinides), α-glucosidase inhibitors, dipeptidyl peptidase-IV (DPP-4) inhibitors, glucagon-like polypeptide-1 (GLP-1), sodium-glucose co-transporter-2 (SGLT2) inhibitors, amylin mimetics, dopamine-2 agonist, and insulin analogues as a second line treatment [6, 7]. Thiazolidinediones (Pioglitazone) is the only third-line treatment of T2DM [8]. The OHAs are, however, heterogeneous in their mode of action that causes intolerable adverse effects and are increasingly failing [9]. Hence, the search for alternative therapies has become the need of the hour.

*Momordica charantia* L. (Family; Cucurbitaceae) has increasingly become alternative therapy for type 2 diabetes mellitus [10]. The plant is distributed widely throughout tropical and subtropical regions and considered native to the African and Australian continents [11]. It is also a vital market vegetable in southern and eastern Asia, and most of East African countries, i.e., Tanzania, Kenya, Uganda, Rwanda, and Burundi [12]. Besides being used as nutritious food, it is a well-known plant in African, Ayurveda, and Chinese traditional systems of medicine for its use in diabetes mellitus.

Interest in the antidiabetic activity of *M. charantia* L. started in the 1940s, where Rivera and colleagues found the hypoglycemic activity of crude extract of *M. charantia* L. in rabbits [13]. Later, several in vivo studies showed significant glucose lowering potential of whole fruits, fruit pulp, and seeds [14–18]. Based on these significant results of in vivo preclinical studies, a handful of clinical trials were conducted [10]; however, the majority of these trials failed to establish benefits of *M. charantia* L. in a systematic review conducted in 2014 [19]. Four years later, a meta-analysis of five trials confirmed significant glucose lowering ability of *M. charantia* L. with only very low certainty of evidence [10]. In this meta-analysis, the researchers observed marked inconsistent results of individual trials and established neither dose nor duration of treatment accurately. These contradictory findings of clinical trials raised a concern about the interpretation and validity of the results of animal models of type 2 diabetes mellitus and their relevance in clinical research.

Our present research aims to assess the efficacy and safety of *M. charantia* L. in preclinical models of type 2 diabetes mellitus. Specifically, this systematic review and meta-analysis will answer the following question; do *M. charantia* L. preparations lower raised blood sugar concentrations in preclinical models of type 2 diabetes mellitus? Such data will provide evidence to improve research practice by identifying risks of bias and study design features that compromise the potential clinical application and contribute to evidence-based clinical trial design.

## Methods

The review authors developed the systematic review and meta-analysis protocol according to the Preferred Reporting Items for Systematic Review and Meta-analysis Protocol Guidelines (PRISMA-P) [20]. They used the PRISMA checklist to ensure completeness of reporting items and optimize the quality of the protocol (see Additional file 1). The authors will report systematic review results according to the PRISMA guidelines, the PRISMA abstract checklist, and guidelines for reporting systematic review and meta-analysis of animal studies [21, 22]. Our protocol registration number is PROSPERO CRD42019119181. Any amendment and reasons for such change to the current protocol will be made public through the PROSPERO database.

### Eligibility criteria

#### Study design eligibility criteria

We will include preclinical studies with a separate control group that assessed the efficacy and safety of *M. charantia* L. treatment. These studies will either be randomized or non-randomized design. We will exclude studies done in a human, in vitro, ex vivo, and in silico study designs. Also, the review will exclude before-after studies without a control group because control groups are necessary to balance baseline variables during the evaluation of the effect of treatment with *M. charantia* L.

#### Animal model eligibility criteria

We will include all in vivo animal models of type 2 diabetes mellitus. The animal models should closely mimic at least some aspects of the pathophysiology of humans with type 2 diabetes mellitus such as insulin resistance and β cell failure to ensure construct validity [23]. All sex, age, strain, and species of animals will be included to ensure adequate clinical generalizability. Type 2 diabetes mellitus may be induced experimentally by chemical, high- fat diet, genetic manipulation, and surgical procedures [24–26]. Table 1 provides an overview of some examples of common preclinical models of type 2 diabetes mellitus. We will exclude animals with endocrinopathies such as hypothyroidism because treatments are likely to be different in these animals.

**Table 1:** Common animal models of type 2 diabetes mellitus

Induction mechanism	Model (species/strains)
Chemically	
i. Streptozotocin	n-STZ, NAD-STZ, STZ-S
ii. Alloxan	Rats, mice, non-primates
iii. Goldthioglucose	Obese diabetic mouse
Genetically	
i. Obese (monogenic)	Lep^ob/ob^ mice, Lepr^db/db^ mice, Zucker diabetic fatty rats
ii. Obese (polygenic)	KK mice, NZO mice, TallyHo/Jng mice NoncNZO10/LtJ mice, TSOD, OLETF rat
iii. Beta cell dysfunction	hIAPP mice, AKITA mice
iv. Spontaneously	Obese rhesus monkey (Macaca mullata)
v. Non-obese (polygenic)	Goto–Kakizaki rats
Obese	
High-fat feeding	C57BL/6J mice, Desert gerbil (*Psammomys obesus*), Nile grass rat (*Arvicanthis niloticus*), Wistar Albino rats
Surgically	
Partial pancreatectomy in animals	Rabbits, diabetic dog model

*OLETF* Otsuka Long-Evans Tokushima Fat rat, *NZO* New Zealand Obese mice, *n-STZ* neonatal streptozotocin-induced diabetes rat, *NAD-STZ* nicotinamide-streptozotocin-induced diabetic, *STZ-S* sucrose-challenged streptozotocin-induced diabetes rat, *KK* Kuo Kundo, *TSOD* Tsumara Suzuki Obese Diabetes mice

#### Intervention

The preclinical intervention group will include animals from studies that evaluated the efficacy or safety of the treatment with *M. charantia* L. preparations (whole extract or fraction of any part of the *M. charantia* L.*)* in any dosing and frequency. The *M. charantia* L. preparations should have been given after the induction of T2DM in animals. Preclinical studies evaluated the efficacy of polyherbal preparations of *M. charantia* L. or isolated pure compounds, concurrent treatment with standard oral hypoglycemic agents, insulin, or any other drug will be excluded because effect size may not be due to *M. charantia* L. alone but partly due to other agents.

#### Comparison

The comparison group will include animals from studies that induced experimental type 2 diabetes mellitus and treated with vehicle. These control groups will facilitate the calculation of effect size and assessment of the safety of the intervention. Healthy animal control will also be used to establish the extent of T2DM induction.

### Information source

The review authors will search MEDLINE through PubMed platform, Web of Science, Embase through Ovid platform, CINAHL, and Scopus. The team will also search gray literature such as conference papers, technical reports, thesis, and dissertations in Google Scholar, Google, OpenGrey, ProQuest Dissertations & Theses, and British Library EThos. The authors will search each database through April 2019. They will also screen reference lists of included studies and reviews for additional eligible studies not retrieved by the search.

#### Search strategy

The search strategy will use a combination of MeSH terms and keywords. The search terms are divided into three components, i.e., the population component which include the words “animals,” “animal,” “animal model,” “preclinical studies,” “experimental animals,” “experimental animal,” “laboratory animal,” “laboratory animals,” “rodents,” “rodent,” “rabbits,” “rabbit,” “rats,” “rat,” “diabetic rats,” “animal disease model,” “mice,” “mouse.” The intervention component with the words “*Momordica charantia*,” “bitter melon,” “bitter gourd,” and “karela.” Finally, the disease component terms will be “diabetes mellitus, type 2,” “non-insulin dependent diabetes mellitus,” “NIDDM,” “glucose metabolic disorders,” “metabolic diseases,” “hyperlipidemia,” “hyperglycemia,” “insulin resistance,” and “glucose intolerance.” The three search components will be combined with the Boolean logic term “AND” while the keywords within each component will be combined with “OR.” The Hooijmans (2010) and de Vries (2011) search filters for the identification of preclinical studies in PubMed and Embase respectively will be applied to increase search efficiency [27, 28]. The authors will not restrict language during the search and identification of studies. The searches will be re-run just before the final analyses to retrieve the most recent studies eligible for inclusion. Additional file 2 provides a more elaborated search strategy applied to PubMed.

### Study records

#### Data management

Review authors will set a weekly update for each database using the generated search strategy. For instance, in PubMed, a group National Center for Biotechnology Information account (NCBI) will be created, and password shared among authors to receive a weekly alert of new articles. Identified articles will be pooled into Mendeley software var. 2.1 (Elsevier). After deduplication, citations will be imported into systematic review facility (SyRF). The SyRF is an online systematic review application for preclinical studies accessible at http://app.syrf.org.uk/home. The review authors will use the online application for screening (titles and abstracts, and full text of the uploaded PDF files), assessing the risk of bias of included studies and data extraction. The extracted data will be stored securely online using SyRF account.

#### Study selection

Titles and abstracts of studies retrieved using the search strategy and those from additional sources will be screened independently by two review authors to identify studies that potentially meet the predetermined inclusion criteria. The full text of these potentially eligible studies will be retrieved and independently assessed for eligibility by two review authors. They will resolve any disagreement between them over the eligibility of particular study through consensus; if no resolution reached, a third reviewer would be involved in the decision.

Further clarification would be sought from the study authors as deemed necessary to determine eligibility. The authors will record reasons for exclusion of each study and report the results of the screening according to the Preferred Reporting Items for Systematic Review and Meta-analysis (PRISMA) flow diagram [29]. Additional file 3 elaborates on each screening stage.

#### Data collection process

Two review authors will extract data independently from the included studies using an online SyRF standardized data extraction application http://app.syrf.org.uk/home. Discrepancies between the reviewers will be identified and resolved through consensus, and a third reviewer will be involved where necessary. Reviewers will contact corresponding authors via email to obtain numerical data if the included study had missing or additional data will be required. The corresponding authors will also be contacted to obtain full text of identified study that had missing abstract or full-text pdf. In case the corresponding authors did not respond to our emails, such incomplete articles will be excluded in our systematic review because data will be collected from full-text articles only. Data presented graphically will be extracted using WebPlotDigitizer; a web-based tool to extract data from plots, images, and maps available at https://automeris.io/WebPlotDigitizer.

### Data items

The extracted data as shown in Table 2 will be used for assessing study quality, evidence synthesis, and safety.

**Table 2:** Data collection items

Domain	Data items
Study characteristics	Study ID, year of publication, the country where the study conducted, sponsorship (funding source)
Study population	Animal species, strain, age, gender, weight, and co-morbidities
T2DM models	Chemical, genetic, surgical, high-fat diet
Intervention and comparison	Taxonomical assessment (scientific name, family, identification of specimen, voucher specimen number), source, part used, description of the preparation, quality control measures, dosage, time of administration, route of administration, and duration of administration
Outcome measures	FPG, HbA1c, IGT, HOMA-IR, HOMA-B, serum insulin concentration, whole pancreas insulin content, morphometrics of pancreases, FFAs, TGs, TC, HDL cholesterol, LDL cholesterol, liver glycogen, ALT, AST, ALP, GGT, urea, BUN, serum creatinine, total protein, albumin, globulin, bilirubin total, calcium, phosphorus, death, Hb, packed cell volume, total red blood cells, total white blood cells, differential white cell counts, platelet count, and absolute red blood cell indices.
Risk of bias assessment and quality of reporting of preclinical study	Baseline data (weight, microbiological status, age, drug- or test-naïve), Sequence generation, allocation concealment, random housing, blinding of investigators/caregivers, random outcome assessment, blinding of the assessor, incomplete outcome data, selective outcome reporting and other source of bias, blinded assessment of outcome, publication after peer review, statement of temperature control, appropriate animal model (aged, diabetic mellitus, type 2), sample size calculation, compliance with animal welfare regulations, statement of potential conflict of interests

*GGT* gamma-glutamyl transpeptidase, *FPG* fasting plasma glucose, *non-FPG* non-fasting plasma glucose, *HbA1*_*c*_ glycosylated hemoglobin A1_c_, *IGT* impaired glucose tolerance, *HOMA-IR* homeostatic model assessment of insulin resistance, *HOMA-B* homeostatic model assessment of β cell function, *FFAs* free fat acids, *TGs* triglycerides, *TC* total cholesterol, *HDL* high-density lipoprotein cholesterol, *LDL* low-density lipoprotein cholesterol, *ALT* alanine aminotransferase, *AST* aspartate aminotransferase, *ALP* alkaline phosphase, *BUN* blood urea nitrogen

### Outcome measures

#### Primary endpoint

The review authors will consider fasting blood glucose concentration (FPG) as a primary endpoint. The blood glucose concentration is the most common end-point for testing therapies in animal models and is clinically the most convenient, cheap, and meaningful indicator of type 2 diabetes mellitus [7].

#### Secondary endpoint

Table 3 indicates key features that associate with type 2 diabetes mellitus and their measurements. This table also highlights the essential safety features which are included for assesment as secondary endpoints. The secondary outcome measures will be collected at baseline and at the end of the follow-up period. However, for glycosylated hemoglobin A1c, only data from studies that have a follow-up of at least 4 weeks will be considered for analysis since this is the minimum time for the treatment to produce meaningful change in glucose control measured by the hemoglobin A1c concentrations [30].

**Table 3:** The secondary endpoint of type 2 diabetes mellitus and their measurement

Features	Measurement
Chronic hyperglycemia	Glycosylated hemoglobin A_1c_
Insulin resistance	HOMA-IR, hyperinsulinaemic–euglycaemic clamp
β cell dysfunction	IGT, HOMA-B, Serum insulin concentration
β cell mass	Whole pancreas insulin content
Lypolysis/obese/dyslipidaemia	FFAs, TGs, TC, HDL cholesterol, LDL cholesterol
Liver glycogen	Liver glycogen
Safety profile	
Weight	Weight of animal
Liver profile	ALT, AST, ALP, Gamma glutamyl transpeptidase
Kidney profile	Urea, urea nitrogen, serum creatinine, total protein, albumin, globulin, bilirubin total
Mortality	Number of animals died*
Ions	Calcium, phosphorus
Hematological parameters	Haemoglobin, packed cell volume, total red blood cells, total white blood cells, differential white cell counts, platelet count, and absolute red blood cell indices

*AST* aspartate aminotransferase, *ALP* alkaline phosphatase, *ALT* alanine aminotransferase, *FFAs* free fat acids, *HDL* high-density lipoprotein cholesterol, *HOMA-IR* homeostatic model assessment of insulin resistance, *HOMA-B* homeostatic model assessment of β cell function, *IGT* impaired glucose tolerance, *LDL* low-density lipoprotein cholesterol, *TC* total cholesterol, *TGs* triglycerides. *Event count, all other outcomes are continuous data

### Risk of bias assessment

Risk of bias for each pre-clinical animal study included will be assessed by SYRCLE’s risk of bias tool [31]. Using the SYRCLE’s risk of bias tool, internal validity of studies will be evaluated through assessment of ten risk of bias domains which are the sequence generation, baseline characteristics, allocation concealment, random housing, blinding of investigators/caregivers, random outcome assessment, blinding of assessor, incomplete outcome data, selective outcome reporting, and other sources of bias. Each criterion will be assigned value as high, low, or unclear risk of bias independently by two reviewers. Discrepancies between the authors will be identified and resolved through consensus, where necessary they may involve a third reviewer.

### Assessment of construct and external validity

Construct validity will be assessed with the extent the experimental models mimic the typical clinical presentation of type 2 diabetes mellitus and experimental operations reflect clinical practice. While external validity will be assessed with the replicability of a cause-effect relationship under different condition such as different models of type 2 diabetes mellitus, geographical conditions, formulations, different investigators, etc. We will use a modified CAMARADES checklist to assess study quality [32]. This checklist is based on 10 criteria: peer-reviewed publication, statement of control of temperature, random allocation to treatment or control, blinded caregiver/investigator, blinded assessment of outcome, use of co-interventions/co-morbid, appropriate animal model (age, sex, species, strain), sample size calculation, compliance with animal welfare regulations, and statement of potential conflict of interests. Each study will be given a quality score out of a possible total of 10 points, and the mean score will be calculated. Studies that will score 1–5 are considered of “low quality” while score 6–10 are considered “high quality”.

### Taxonomical assessment of included studies

The taxonomical and nomenclatural accuracy will be assessed by comparing reported taxonomical information with existing standards in open botanical database accessible at www.theplantlist.org. Frequency of erroneous names use, types of such errors, identification of a specimen, and voucher specimen deposited will be assessed according to methods proposed by Rivera and colleagues [33]. The review authors will give grade “A” for studies with full information about the species of plant, identification of the specimen, and voucher specimen deposited. While they will give grade “B” for those with partial information about the species of plant such as an identification of specimen and a voucher specimen not presented and inaccurate taxonomic information, finally grade “C” for studies with inadequate information about the species of plant, or an identification of specimen and a voucher specimen were not presented at all.

### Strategy for data synthesis

*Qualitative analysis*: Data from eligible studies will be described in a narrative synthesis. The narrative synthesis will summarize study characteristics, population (animals), type of models of type 2 diabetes mellitus used, intervention, and comparison studied in textual form.

*Quantitative analysis*: Quantitative data will be pooled in a statistical meta-analysis using Review Manager (RevMan) software 5.3 (Copenhagen: The Nordic Cochrane Centre, The Cochrane Collaboration, 2014). The meta-analysis will only be conducted when there are two or more studies that had data on particular outcome of interest [34]. These outcomes include FPG, HbA1c, IGT, HOMA-IR, HOMA-B, serum insulin concentration, whole pancreas insulin content, FFAs, TGs, TC, HDL cholesterol, LDL cholesterol, liver glycogen, ALT, AST, ALP, GGT, urea, BUN, serum creatinine, total protein, albumin, globulin, bilirubin total, calcium, phosphorus, hemoglobin, packed cell volume, total red blood cells, total white blood cells, differential white cell counts, platelet count, and absolute red blood cell indices will be analyzed in meta-analysis. Since the outcomes of interest are continuous variables, authors will use the standardized mean difference (SMD) to evaluate the effect of *M. charantia* L. In this method, the difference in means between intervention and control groups at follow-up will be divided by pooled standard deviation of the two groups to convert all outcome measures to standardized scale with a unit of standard deviation. Animal experimental studies are generally considered to have a small sample size of fewer than ten animals per group [35]; hence, Hedge’s G effect sizes will be used for calculating SMD [36]. The inverse variance-weighted method will be used to attribute the relative contribution of each included study to pooled SMD effect of *M. charantia* L. and its 95% confidence intervals [37]. Random effect model will be used for pooling effect estimates because reviewers believe that the effect sizes from animal studies are more likely to differ due to the difference in design characteristics. For dichotomous data (mortality), effect sizes will be expressed as odds ratios and 95% confidence intervals incorporating a random effects modeling approach.

#### Heterogeneity assessment

Cochran’s Q will be used to assess heterogeneity [38]. Using this test, it is assumed that studies are drawn from the same animal population and measure the same thing. Thus, Cochran’s Q can be tested using a chi-squared (*χ*^2^) test and its *P* value to evaluate heterogeneity between primary studies intervention effects. A low *P* value (or a large *χ*^2^ statistic relative to its degree of freedom) provides evidence that the observed variation in estimates of effect is not due to chance alone. However, a non-significant *P* value does not necessarily indicate absence of heterogeneity because few comparisons and small sample size as always the case in animal studies usually contribute to false results. We will therefore use additional measure; *I*^2^ statistic for assessing heterogeneity severity as this statistic does not depend on the number of comparisons in meta-analysis [39]. The *I*^2^ of 75 or more will be considered as indicative of substantial heterogeneity [40, 41].

Sub-group analysis will be used to examine potential variables that might explain heterogeneity on primary outcome (FPG). When there are at least ten studies per sub-group, authors will use the sub-group analysis to compare effect sizes of categorical variables [39]. The potential variables of interest are risk of bias score (high risk, low risk score), study design (randomized and non-randomized design), duration of treatment (≤ 1 month versus > 1 month), dose (different dose groups), nature of intervention (aqueous extract, alcoholic extract) , animal species (mouse, rat, rabbit, dog), animal strains (KK mice, C57BL/6 J mice, others), animal age (young versus older), sex (male, female) and model of induction of type 2 diabetes mellitus (chemical, genetic, surgical, high-fat diet). The sub-group analysis is based on analysis of variance assumes the between-study variance (*τ*^2^) to be the same in all subgroups. To account for potential false positive due to multiple comparisons, a Bonferroni correction to control familywise error rate will be used. In this test, a familywise error rate (0.05) will be devided by a number of tests to obtain adjucted *P* value for an individual test [42].

#### Publication bias

Publication bias for each outcome will be assessed by testing the asymmetry of the funnel plot using Egger’s test [43, 44]. The test for funnel plot asymmetry will not be used when there are fewer than ten primary studies in the meta-analysis because test power is generally too low to distinguish chance from real asymmetry [45]. If publication bias is significant, trim and fill method will be used to correct the probable publication bias. Also, the significant asymmetry of the funnel plot will be interpreted in the context of susceptibility to other biases that might explain it.

#### Assessment of confidence in cumulative evidence

Review authors will use “The Grading of Recommendations, Assessment, Development, and Evaluation (GRADE) approach” as a framework to rate the certainty in the evidence of preclinical animal studies [46, 47]. The rating of certainty will be done for each outcome by considering the risk of bias (as assessed by SYCLE’s RoB tool), inconsistency (as assessed by heterogeneity tests, confidence intervals, and *P* values), imprecision, publication bias, and indirectness [47]. The indirectness will be assessed by considering how well the animal studies represent clinical population, intervention, comparator, and outcome (PICO). We will upgrade stating rate of low-quality evidence to one level if there is a large magnitude of the effect, presence of dose-response relationship, and opposing the direction of plausible residual confounding. After considering all factors, evidence will be finally rated as high, moderate, low, or very low-quality evidence and results presented as summary of findings table.

### Knowledge translation

Findings of this systematic review will be of interest to several groups. Firstly, to reach the broader scientific community, a manuscript will be developed and published in a peer review journal prominent in this field. Secondly, authors will attend and present systematic review results in both national and international scientific conferences. Lastly, the authors will also present the findings to groups of MSc and Ph.D. students at Pharm-BioTechnology and Traditional Medicine Centre of Excellence, Mbarara University of Science and Technology, Uganda, and Institute of traditional Medicine, Muhimbili University of Health and Allied Sciences, Tanzania. These students are working on natural products development that involves the use of animals. Published results will be widely disseminated through professional media such as researchgate, Twitter, and LinkedIn for wider potential knowledge users.

## Discussion

The present work provides a protocol for systematic review and meta-analysis of preclinical studies that investigated the efficacy and safety of *M. charantia* L. on animal models of type 2 diabetes mellitus. The results of this study will be useful to clinical researchers and herbal practitioners in the treatment of type 2 diabetes mellitus. It could be interpreted that if adequate evidence of efficacy from preclinical studies is established, it could mean the preclinical models of type 2 diabetes mellitus were not sufficient to predict clinical efficacy of *M. charantia* L. preparations. On the other hand, lack of adequate evidence of efficacy could mean previous clinical trials which failed were conducted based on insufficient preclinical evidence. Such interpretations will carefully consider various factors influence translating animal data to the clinical practice, such as biological differences between species, internal validity, differences in experimental design between animal studies and clinical trials, insufficient reporting, and publication bias [48].

Our systematic review is timely because there has been a growing number of preclinical and clinical studies investigating the efficacy and underlying mechanism of action of *M. charantia* L. in lowering elevated blood sugar level [16, 49, 50]. Such studies could benefit from the results of the systematic review and improve design features identified to compromise the potential clinical application. It is also likely that this systematic review will contribute to the implementation of the reduction, refinement, and replacement (3Rs) in animal studies of *M. charantia* L. [51]. If the results indicate that there are sufficient preclinical evidences that *M. charantia* L. is effective in lowering elevated blood sugar, authors could recommend no further animal studies is required and thus reduce number of animals used.

## Supplementary information

**Additional file 1.** PRISMA-P 2015 Checklist

**Additional file 2.** Search strategy for PubMed

**Additional file 3.** PRISMA flow diagram for study inclusion

## Data Availability

The datasets generated and/analyzed during the current study will be available from the corresponding author according to the institution guideline on materials and data transfer agreements.

## Abbreviations

ACE-II: The Eastern and Southern Africa Higher Education Centers of Excellence Project
ADA: American Diabetes Association
ALP: Alkaline phosphatase
ALT: Alanine aminotransferase
AST: Aspartate aminotransferase
CAMARADES: Collaborative Approach to Meta-Analysis and Review of Animal Data from Experimental Studies
DPP-4: Dipeptidyl peptidase-IV
FFAs: Free fat acids
GLP-1: Glucagon-like peptide-1
HbA1c: Glycosylated hemoglobin A1c
HDL: High-density lipoprotein cholesterol
HOMA-IR: Homeostatic model assessment of β cell insulin resistance
IGT: Impaired glucose tolerance
LDL: Low-density lipoprotein cholesterol
NIDDM: Non-insulin dependent diabetes mellitus
OHAs: Oral hypoglycemic agents
PHARMBIOTRAC: Pharm-BioTechnology and Traditional Medicine Center of Excellence
PRISMA: Preferred Reporting Items for Systematic Review and Meta-analysis
PRISMA-P: Preferred Reporting Items for Systematic Review and Meta-analysis Protocols
SAFV: Simple analysis of final values
STREAM: Studies of Translation, Ethics, and Medicine
STZ: Streptozotocin
SYRCLE: Systematic Review Centre for Laboratory Animal Experimentation
T2DM: Type 2 diabetes mellitus
