# Supplementary material for: Systematic review and meta-analysis protocol for efficacy and safety of Momordica charantia L. on animal models of type 2 diabetes mellitus
Source: Syst Rev. 2020 Jan 8;9:7. doi: 10.1186/s13643-019-1265-4 (PMC6950794; doi:10.1186/s13643-019-1265-4)
Supplement: Supplementary file 3 — Additional file 3. PRISMA flow diagram for study inclusion [file 13643_2019_1265_MOESM3_ESM.docx]

**Additional file 3**

**PRISMA flow diagram for study inclusion**

Studies included in quantitative synthesis (meta-analysis)
(n = )

Studies included in qualitative synthesis
(n = )

Full-text articles excluded, with reasons
(n = )

Full-text articles assessed for eligibility
(n = )

Records excluded
(n = )

Records screened
(n = )

Records after duplicates removed
(n = )

Additional records identified through other sources
(n = )

Records identified through database searching
(n = )

## Identification

## Screening

## Eligibility

## Included

**Fig. 1. PRISMA flow diagram for study inclusion**
